# Supplementary material for: Splice-Junction-Based Mapping of Alternative Isoforms in the Human Proteome
Source: Cell Rep. Author manuscript; Available in PMC 2020 Jan 15. (PMC6961840; doi:10.1016/j.celrep.2019.11.026)
Supplement: 2 [file NIHMS1546469-supplement-2.zip › DF1/PXD009737/TestisGluC/TestisGluC_4_PARK7_MRRAGGPYDVVVLPGGNLGAQNLSE.pdf]

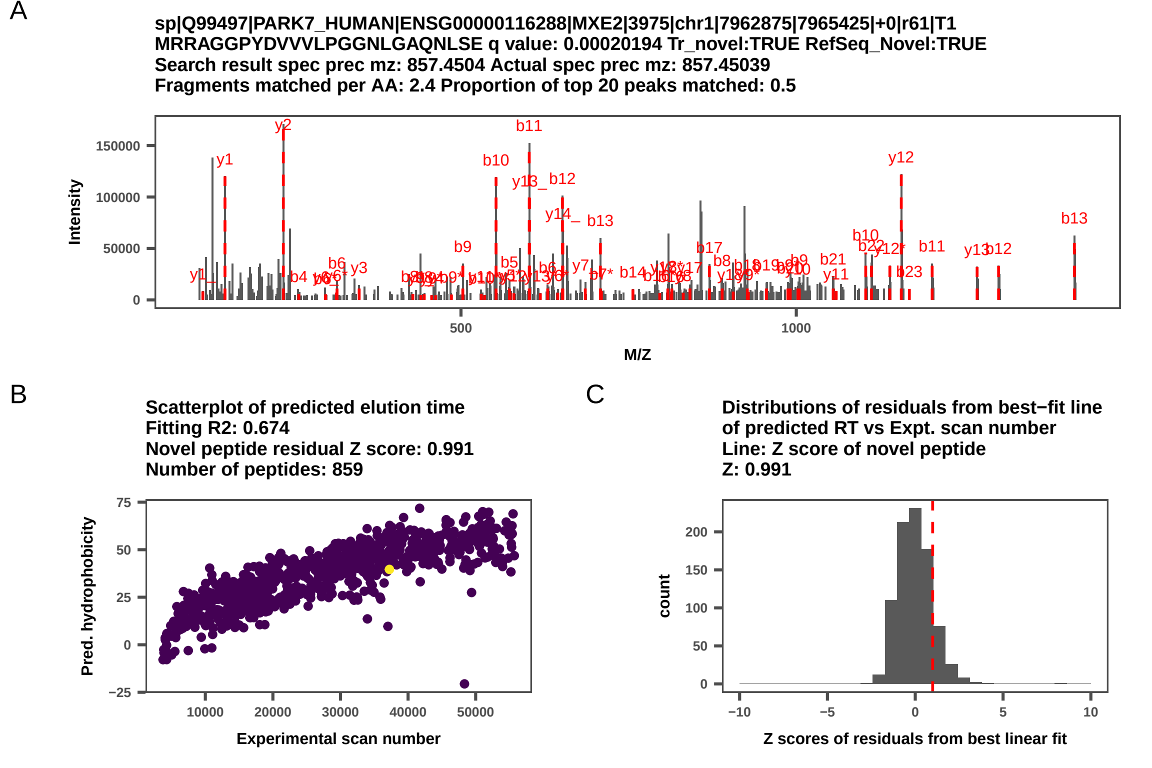
A
sp|Q99497|PARK7_HUMAN|ENSG00000116288|MXE2|3975|chr1|7962875|7965425|+0|r61|T1
MRRAGGPYDVVVLPGGNLGAQNLSE q value: 0.00020194 Tr_novel:TRUE RefSeq_Novel:TRUE
Search result spec prec mz: 857.4504 Actual spec prec mz: 857.45039
Fragments matched per AA: 2.4 Proportion of top 20 peaks matched: 0.5
Intensity
150000
100000
50000
0
500
1000
M/Z
y1
y2
b10
b11
b12
b13
y12
b9
b13
B
Scatterplot of predicted elution time
Fitting R2: 0.674
Novel peptide residual Z score: 0.991
Number of peptides: 859
Pred. hydrophobicity
75
50
25
0
−25
10000
20000
30000
40000
50000
Experimental scan number
C
Distributions of residuals from best−fit line
of predicted RT vs Expt. scan number
Line: Z score of novel peptide
Z: 0.991
count
200
150
100
50
0
−10
−5
0
5
10
Z scores of residuals from best linear fit
